# Editorial Notes

**Published:** 1939

**Authors:** 


### Editorial Notes

**Opening of Barrow Hospital.**

On 3rd May the new Barrow Hospital, built by the Visiting Committee of the Bristol Mental Hospital was opened by the Chairman of the Board of Control, Sir Laurence Brock, C.B. The need for additional accommodation for mental patients had been realized for many years, until at last a site was purchased at Barrow Gurney, and the City Council, on 10th October, 1933, approved the scheme for building the new hospital. The site lies only five miles from the centre of the city in a woodland area, picturesquely named "The Wild Country." The plans for the hospital were prepared by Sir George Oatley in consultation with the Board of Control and Dr. E. Barton White, who was at that time Medical Superintendent of the Bristol Mental Hospital. By kind permission of the Chairman, Alderman Mrs. Pheysey, J.P., and the members of the Visiting Committee, part of Dr. Barton White's description of the new hospital is quoted from the booklet published for the opening ceremony :—

Since the time of Hippocrates, history reveals only sporadic attempts at breaking away from superstition in the treatment of disease affecting the mind until comparatively recent times, and it was not until during the French Revolution that certain reforms were introduced both in this country and in France.

Early in the nineteenth century there were two outstanding instances in this country of advances in the methods of treatment and nursing which have since been followed by the majority, and many of these reforms have since become compulsory by law. Space prohibits giving here the full history of progress made in Bristol, but records dating from 1840 refer to the " pens " and the " yard " attached to St. Peter's Hospital, that picturesque old building in the centre of the city, where the " yard," or small exercising ground, was protected from the harbour by spiked railings.

The hospital at Fishponds was built in 1860 for some two hundred patients with occasional extensions up to one thousand and forty-five by the year 1908, but by 1935 the number resident had exceeded eleven hundred, coincident with the growth of the city. In 1932 a modern Nurses' Home and a Medical Officer's House were completed, and in February of the same year the Home and Out-patient Clinic at Grove Road received its first patient.

Most of the older hospitals still in use were built like large barracks, under one roof, and though comfortable in some respects, they provided little relief to those sensitive to their environment, a problem now recognized as so important a part of successful treatment, and there were no adequate means for proper classification. This became well nigh impossible with increasing numbers and it was difficult to carry out any form of treatment.

To those who have spent many years amongst their patients it became evident that hospitals built on the villa system, breaking away from the institution atmosphere, are essential for the realisation of satisfactory results, where the environment is peaceful and conducive to rest and contentment, and where there is full scope for proper classification. Several such hospitals now exist, and the opportunity to provide these conditions arose in Bristol when additional accommodation became necessary owing to the increasing population and consequent rise in the number of admissions to the hospital at Fishponds.

The principle upon which this hospital was conceived is that the Admission Centre is the first part of the hospital to be approached on entering the estate.

In this Admission and Treatment Centre the early and acute phase of the illness is dealt with in all its aspects and full investigations are made and recorded. When considered well enough, patients are passed on to the Convalescent Villas and further to the Parole Villas. From any of these units patients may be discharged upon recovery, while the well-equipped Sick Hospital provides treatment for those suffering from any intercurrent bodily illness at any time during their stay.

There are facilities for all forms of occupation therapy, and, as in most hospitals of this kind, provision is made for Farm buildings, and there is both horticultural and agricultural land upon which patients can be usefully employed with benefit to themselves.

The whole lay-out is so arranged that most of the buildings are well screened from one another, and the Architect is to be congratulated upon the effect produced.

The project is intended for an ultimate total of 1,150 patients. Its execution has been divided into stages. The initial buildings accommodate 375 patients with Residential Quarters for medical and other officers and nurses.

**Burden Neurological Institute.**

On 12th May Sir Thomas Inskip, Secretary of State for the Dominions, inaugurated the Burden Neurological Institute, the latest of the many benefactions of Mrs. R. G. Burden, of Clevedon Hall, and Warden of Stoke Park Colony.

Sir Laurence Brock, C.B., Chairman of the Board of Control, who is also Chairman of the Committee of Management of the new Neurological Institute, presided at the ceremony, at which the donor, Mrs. Burden, was present.

This Institute is the most recent development of a series of activities associated with Mrs. Burden and her husband. It is not directly concerned with mental deficiency, and although it is situated in Stoke Park it does not form part of the colony for mental defectives which has been so long established there. The Director of the Institute is Dr. F. L. Golla, who was associated with the late Sir Victor Horsley and Sir F. W. Mott. Since Mott's death, and for a short time previously, he has been Director of the Central Pathological Laboratory of the London County Council. The Institute is to receive financial assistance from the Rockefeller

Foundation in America and the Medical Research Council in Britain. The purpose and aim of the Institute was described in the *Western Daily Press* of 12th May, as being to undertake an intensive study of the chemical and physical processes of the body in certain nervous mental disorders with special concentration on epilepsy.

The immediate task of the Institute will be to investigate a few carefully selected cases by every form of bio-chemical, electro-physiological and endocrinological method.

Of Dr. Golla's fitness to direct these studies there can be no doubt, and we welcome him as a colleague as warmly as we do the foundation of the Burden Neurological Institute.

**Colston Research Society.**

THE Thirty-Fifth Annual Dinner of the Colston Research Society was held on 25th May. The guest of the evening was Mr. Joseph P. Kennedy, the American Ambassador, whose health was proposed by Mr. Winston Churchill, the Chancellor of the University. A matter of great regret was the absence, through illness, of the President, Mr. Foster G. Robinson. The immediate Past President, Mr. R. J. Sinclair, presided in his place. Nearly 400 guests attended the dinner, and, in the words of the Vice-Chancellor, "it was the most distinguished night in the history of the Society." The collection was announced as £781 14s. 6d. A particularly gratifying consequence of the large attendance was the addition of forty-five new names to the Society's subscription list, which augurs well for the future.

